# Supplementary material for: Application of Transcriptome Analysis to Understand the Adverse Effects of Hypotonic Stress on Different Development Stages in the Giant Freshwater Prawn Macrobrachium rosenbergii Post-Larvae
Source: Antioxidants (Basel). 2022 Feb 22;11(3):440. doi: 10.3390/antiox11030440 (PMC8944765; doi:10.3390/antiox11030440)
Supplement: Supplementary file 1 [file antioxidants-11-00440-s001.zip › Supplementary Materials1.pdf]

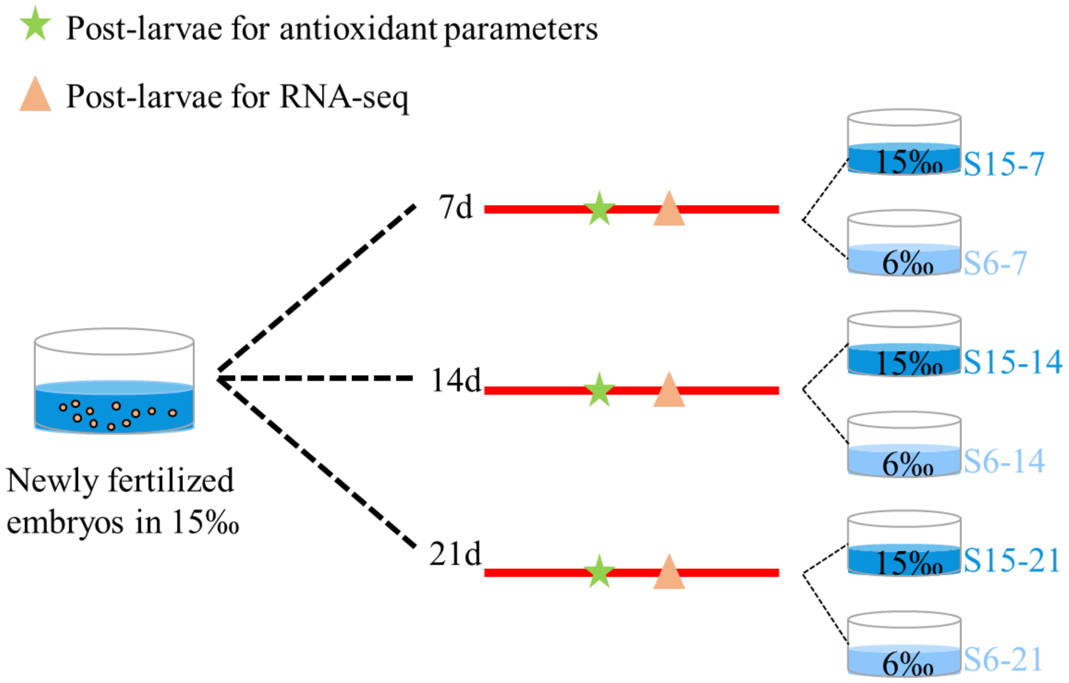

Figure S1. The experimental design and sampling scheme of this study.

Acclimation at two salinities (6‰, 15‰) for three development stages. Since embryos fertilized in normal concentration (15‰). Then the collection of corresponding tissues for RNA sequencing and antioxidant parameters.
