# Supplementary material for: Application of Transcriptome Analysis to Understand the Adverse Effects of Hypotonic Stress on Different Development Stages in the Giant Freshwater Prawn Macrobrachium rosenbergii Post-Larvae
Source: Antioxidants (Basel). 2022 Feb 22;11(3):440. doi: 10.3390/antiox11030440 (PMC8944765; doi:10.3390/antiox11030440)
Supplement: Supplementary file 1 [file antioxidants-11-00440-s001.zip › Supplementary Materials2.pdf]

Table S1. Primers used in the study.

Genes	Primer sequences (5′-3′)	Product size (bp)
ATG13 (F)	AGACCAAGAAGGGCTTAGGAGG	126
ATG13 (R)	CATCTATAATTGCGAGCGTCCA	
ATG3 (F)	GCCGATGGAACTGATGAAGACG	141
ATG3 (R)	ACTAGAGGCTGCTGCTGGGAT	
Barkor (F)	AACCAGCCAGAAGTATTGACGC	156
Barkor (R)	AACCAGGCAAAAGAGGATGAAC	
ATG7 (F)	GATGGGACCTCGCATGGTAA	159
ATG7 (R)	CCAGCCCCTAAAAGCAGACA	
Beclin (F)	TTGCCGAACTCGCTCTACCG	166
Beclin (R)	GGCTGAGTTTTCTCCGTGTCGT	
NRBF2 (F)	AGCAAGCAACGGGAGAAGTATG	101
NRBF2 (R)	CTTTTGGACGGGTGAGCAGTA	
VMP1 (F)	GGGGCAACGACTCAGGATGA	119
VMP1 (R)	TTGTGGTAGCTCTGGGCAAAA	
ATG2 (F)	TCGCCGCAATCAGTTACCA	142
ATG2 (R)	TTGAATGCCCCTCACTACTCG	
beta-actin (F)	GGCATTCACGAGACCACTTACA	185
beta-actin (R)	TCGGGAGGTGCGATGATTTT	
