# Supplementary material for: Application of Transcriptome Analysis to Understand the Adverse Effects of Hypotonic Stress on Different Development Stages in the Giant Freshwater Prawn Macrobrachium rosenbergii Post-Larvae
Source: Antioxidants (Basel). 2022 Feb 22;11(3):440. doi: 10.3390/antiox11030440 (PMC8944765; doi:10.3390/antiox11030440)
Supplement: Supplementary file 1 [file antioxidants-11-00440-s001.zip › Supplementary Materials3.pdf]

Table S2. Quality control and data statistics for clean reads.

Sample	Raws Reads	Clean Reads	Clean Ratio (%)	Reads Mapped	Mapping Ratio (%)	Clean Q30 (%)	Clean GC (%)
S15-7-1	54174512	50424296	93.08	28761402	57.04	94.20	48.08
S15-7-2	44664868	40939394	91.66	23814950	58.17	91.04	49.72
S15-7-3	49419690	45681845	92.44	26288176	57.61	92.62	48.90
S15-14-1	45910602	41159624	89.65	24472746	59.46	92.44	49.53
S15-14-2	46433974	42378468	91.27	24756212	58.42	91.53	48.00
S15-14-3	46172288	41769046	90.46	24614479	58.94	91.99	48.77
S15-21-1	37317656	32538180	87.19	23159374	71.18	91.33	52.94
S15-21-2	42917192	33839366	78.85	20774512	61.39	92.03	49.89
S15-21-3	40117424	33188773	82.73	21966943	66.29	91.68	51.42
S6-7-1	49787776	46411224	93.22	27360974	58.95	94.32	47.01
S6-7-2	60433074	56567850	93.60	34090710	60.27	94.50	46.83
S6-7-3	55110425	51489537	93.43	30725842	59.61	94.41	46.92
S6-14-1	55046066	44655876	81.12	27530732	61.65	92.75	46.02
S6-14-2	51561910	43314186	84.00	26175974	60.43	91.92	48.48
S6-14-3	53303988	43985031	82.52	26853353	61.04	92.34	47.25
S6-21-1	34119258	28259706	82.83	17775644	62.90	92.14	49.66
S6-21-2	49575820	42177322	85.08	25130566	59.58	91.66	48.31
S6-21-3	41847539	35218514	84.16	21453105	61.24	91.90	48.99

Note: S15-7: 7 day-post-hatch larvae in 15‰ salinity; S15-14: 14 day-post-hatch larvae in 15‰ salinity; S15-21: 21 day-post-hatch larvae in 15‰ salinity; S6-7: 7 day-post-hatch larvae in 6‰ salinity; S6-14: 14 day-post-hatch larvae in 6‰ salinity; S6-21: 21 day-post-hatch larvae in 6‰ salinity.
